# Refining prognosis in advanced renal cell carcinoma: international real-world validation of the Meet-URO score in first-line immunotherapy combinations

**DOI:** 10.1093/oncolo/oyag203

**Published:** 2026-05-20

**Authors:** Sara Elena Rebuzzi, Aruni Ghose, Sarah Rudman, Balaji Venugopal, Kate Young, Ricky Dylan Frazer, Olubukola Ayodele, Mark Stares, Brent O’Carrigan, Waqas Ali, John McGrane, Ankit Jain, Ondrej Fiala, Vishwani Chauhan, Agnieszka Michael, Anjali Zarkar, Gaurav Kapur, Natalie Charnley, Mehran Afshar, Salil Vengalil, Caroline Forde, Janet Brown, Yüksel Ürün, Diletta Bianchini, Amit Bahl, Narayanan Srihari, Fabrizio Di Costanzo, Benjamin Smalley, Joanne Parkes, Simon Crabb, Naveen Vasudev, Alexandr Poprach, Nicholas Brown, Lijo James, Sophia Haywood, Jose Tapia, Anupama Vijay, Jane Parry, Michael Cheung, Ishika Mahajan, Niall O Moon, Ritika Abrol, Michaela Tkadlecová, Yamin Shwe Yee Soe, Anum Zargham, Michelle Smith, Sophie Ashley, Orla Hardy, Grisma Patel, Kyaw Kyaw Tun, Emma Johnston, Abdullah Sarwer, Hatice Bölek, Roshani Shrestha, Amarnath Challapalli, James Meegan, Shobana Anpalakhan, Francesco Buono, Eva Kolarikova, David Ka-Wai Leung, Veronica Murianni, Fabio Catalano, Davide Bimbatti, Sebastiano Buti, Alessio Signori, Giuseppe Fornarini, Pasquale Rescigno, Jeremy Yuen Chun Teoh, Giuseppe Luigi Banna

**Affiliations:** Medical Oncology Unit 2, Ospedale Molinette, Azienda Ospedaliero-Universitaria Città della Salute e della Scienza di Torino, Torino, 10126, Italy; Barts Cancer Centre, Barts Health NHS Trust, London, EC1A 7BE, United Kingdom; Cancer Centre at Guy’s, Guy’s and St Thomas’ NHS Foundation Trust, London, SE1 9RT, United Kingdom; Beatson West of Scotland Cancer Centre, NHS Greater Glasgow and Clyde, Glasgow, G12 0YN, United Kingdom; Department of Medical Oncology, The Royal Marsden NHS Foundation Trust, London, SW3 6JJ, United Kingdom; Velindre Cancer Centre, Velindre University NHS Trust, Cardiff, CF14 2TL, United Kingdom; Leicester Cancer Research Centre, University Hospitals of Leicester NHS Trust, Leicester, LE1 5WW, United Kingdom; Edinburgh Cancer Centre, NHS Lothian, Western General Hospital, Edinburgh, EH4 2XU, United Kingdom; Department of Oncology, Cambridge University Hospitals NHS Foundation Trust, Cambridge, CB2 0QQ, United Kingdom; Department of Oncology, United Lincolnshire Hospitals NHS Trust, Lincoln, LN2 5QY, United Kingdom; Sunrise Oncology Centre, Royal Cornwall Hospitals NHS Trust, Cornwall, TR1 3LJ, United Kingdom; Deanesly Centre for Cancer Services, The Royal Wolverhampton NHS Trust, Wolverhampton, WV10 0QP, United Kingdom; Department of Oncology and Radiotherapeutics, Faculty of Medicine, University Hospital in Pilsen, Charles University Prague, 323 00, Czech Republic; Barts Cancer Centre, Barts Health NHS Trust, London, EC1A 7BE, United Kingdom; Royal Surrey Cancer Centre, Royal Surrey NHS Foundation Trust, Guildford, GU2 7XX, United Kingdom; Department of Oncology, University Hospitals Birmingham NHS Foundation Trust, Birmingham, B15 2GW, United Kingdom; Department of Oncology, Norfolk and Norwich University Hospitals NHS Foundation Trust, Norwich, NR4 7UY, United Kingdom; Rosemere Cancer Centre, Lancashire Teaching Hospitals NHS Foundation Trust, Preston, PR2 9HT, United Kingdom; Department of Oncology, St George’s University Hospitals NHS Foundation Trust, London, SW17 0QT, United Kingdom; Cancer Centre, Royal Stoke University Hospital, University Hospital of North Midlands NHS Trust, Stoke on Trent, ST4 6QG, United Kingdom; Northern Ireland Cancer Centre, Belfast Health and Social Care Trust, Belfast, BT9 7JL, United Kingdom; Weston Park Cancer Centre, Sheffield Teaching Hospitals NHS Foundation Trust, Sheffield, S10 2SJ, United Kingdom; Department of Medical Oncology, Ankara University Cancer Research Institute, Ankara, 06590, Turkey; Kent Oncology Centre, Maidstone and Tunbridge Wells NHS Trust, Maidstone, ME16 9QQ, United Kingdom; Bristol Haematology and Oncology Centre, University Hospitals Bristol NHS Foundation Trust, Bristol, BS2 8ED, United Kingdom; Lingen Davies Cancer Centre, Shrewsbury and Telford Hospital NHS Trust, Shrewsbury, SY3 8XQ, United Kingdom; Translational and Clinical Research Institute, Centre for Cancer, Newcastle University, Newcastle upon Tyne, NE2 4HH, United Kingdom; Medical Oncology, University of Naples Federico II, Naples, 80131, Italy; Department of Oncology, Portsmouth Hospitals University NHS Trust, Portsmouth, PO6 3LY, United Kingdom; Faculty of Science and Health, School of Pharmacy and Biomedical Sciences, University of Portsmouth, Portsmouth, PO1 2DT, United Kingdom; Worcestershire Oncology Centre, Worcestershire Acute Hospitals NHS Trust, Worcester, WR5 1DD, United Kingdom; Department of Oncology, University Hospital Southampton NHS Foundation Trust, Southampton, SO16 6YD, United Kingdom; Leeds Cancer Centre, Leeds Teaching Hospitals NHS Trust, Leeds, LS9 7TF, United Kingdom; Department of Comprehensive Cancer Care and Faculty of Medicine, Masaryk Memorial Cancer Institute and Masaryk University, Brno, 656 53, Czech Republic; Cancer Centre at Guy’s, Guy’s and St Thomas’ NHS Foundation Trust, London, SE1 9RT, United Kingdom; Beatson West of Scotland Cancer Centre, NHS Greater Glasgow and Clyde, Glasgow, G12 0YN, United Kingdom; Department of Medical Oncology, The Royal Marsden NHS Foundation Trust, London, SW3 6JJ, United Kingdom; Velindre Cancer Centre, Velindre University NHS Trust, Cardiff, CF14 2TL, United Kingdom; Leicester Cancer Research Centre, University Hospitals of Leicester NHS Trust, Leicester, LE1 5WW, United Kingdom; Edinburgh Cancer Centre, NHS Lothian, Western General Hospital, Edinburgh, EH4 2XU, United Kingdom; Department of Oncology, Cambridge University Hospitals NHS Foundation Trust, Cambridge, CB2 0QQ, United Kingdom; Department of Oncology, United Lincolnshire Hospitals NHS Trust, Lincoln, LN2 5QY, United Kingdom; Sunrise Oncology Centre, Royal Cornwall Hospitals NHS Trust, Cornwall, TR1 3LJ, United Kingdom; Deanesly Centre for Cancer Services, The Royal Wolverhampton NHS Trust, Wolverhampton, WV10 0QP, United Kingdom; Department of Oncology and Radiotherapeutics, Faculty of Medicine, University Hospital in Pilsen, Charles University Prague, 323 00, Czech Republic; Royal Surrey Cancer Centre, Royal Surrey NHS Foundation Trust, Guildford, GU2 7XX, United Kingdom; Department of Oncology, University Hospitals Birmingham NHS Foundation Trust, Birmingham, B15 2GW, United Kingdom; Department of Oncology, Norfolk and Norwich University Hospitals NHS Foundation Trust, Norwich, NR4 7UY, United Kingdom; Rosemere Cancer Centre, Lancashire Teaching Hospitals NHS Foundation Trust, Preston, PR2 9HT, United Kingdom; Department of Oncology, St George’s University Hospitals NHS Foundation Trust, London, SW17 0QT, United Kingdom; Department of Oncology, St George’s University Hospitals NHS Foundation Trust, London, SW17 0QT, United Kingdom; Cancer Centre, Royal Stoke University Hospital, University Hospital of North Midlands NHS Trust, Stoke on Trent, ST4 6QG, United Kingdom; Northern Ireland Cancer Centre, Belfast Health and Social Care Trust, Belfast, BT9 7JL, United Kingdom; Weston Park Cancer Centre, Sheffield Teaching Hospitals NHS Foundation Trust, Sheffield, S10 2SJ, United Kingdom; Department of Medical Oncology, Ankara University Cancer Research Institute, Ankara, 06590, Turkey; Kent Oncology Centre, Maidstone and Tunbridge Wells NHS Trust, Maidstone, ME16 9QQ, United Kingdom; Bristol Haematology and Oncology Centre, University Hospitals Bristol NHS Foundation Trust, Bristol, BS2 8ED, United Kingdom; Lingen Davies Cancer Centre, Shrewsbury and Telford Hospital NHS Trust, Shrewsbury, SY3 8XQ, United Kingdom; Department of Oncology, University Hospital Southampton NHS Foundation Trust, Southampton, SO16 6YD, United Kingdom; Leeds Cancer Centre, Leeds Teaching Hospitals NHS Trust, Leeds, LS9 7TF, United Kingdom; Department of Comprehensive Cancer Care and Faculty of Medicine, Masaryk Memorial Cancer Institute and Masaryk University, Brno, 656 53, Czech Republic; Department of Surgery, S.H. Ho Urology Centre, The Chinese University of Hong Kong, Hong Kong, China; S.C. Oncologia, Ospedale “Borea” di Sanremo, ASL1 Imperiese, Sanremo, 18038, Italy; S.C. Oncologia, Ospedale “Borea” di Sanremo, ASL1 Imperiese, Sanremo, 18038, Italy; Oncology Unit 1, Istituto Oncologico Veneto—IOV IRCCS, Padova, 35128, Italy; Medical Oncology Unit, University Hospital of Parma, Parma, 43126, Italy; Department of Medicine and Surgery, University of Parma, Parma, 43125, Italy; Department of Health Sciences (DISSAL), Section of Biostatistics, University of Genova, Genova, 16132, Italy; Medical Oncology Unit 1, IRCCS Ospedale Policlinico San Martino, Genova, 16132, Italy; Translational and Clinical Research Institute, Centre for Cancer, Newcastle University, Newcastle upon Tyne, NE2 4HH, United Kingdom; Department of Surgery, S.H. Ho Urology Centre, The Chinese University of Hong Kong, Hong Kong, China; Faculty of Science and Health, School of Pharmacy and Biomedical Sciences, University of Portsmouth, Portsmouth, PO1 2DT, United Kingdom

**Keywords:** metastatic renal cell carcinoma, prognostic, score, immunotherapy, immune-combinations, first line

## Abstract

**Background:**

Effective risk stratification is essential for guiding treatment decisions in patients with metastatic renal cell carcinoma (mRCC). The Meet-URO score is a novel prognostic model that integrates the International Metastatic RCC Database Consortium (IMDC) criteria with neutrophil-to-lymphocyte ratio (NLR) and the presence of bone metastases. Developed in the immunotherapy era, it has demonstrated superior prognostic accuracy compared to the IMDC score across various clinical settings and treatment strategies. Its validation in the context of first-line immune-based combinations has been awaited.

**Methods:**

External validation of Meet-URO was performed using a large retrospective real-world cohort of mRCC patients treated with first-line immune-based combinations. Secondary analyses included a comparison with the IMDC score for predicting overall survival (OS) and progression-free survival (PFS). Additionally, restricted mean survival time (RMST) was assessed.

**Results:**

A total of 1,418 patients were included in the analysis: 54% received ICI–ICI regimen (nivolumab plus ipilimumab), while 46% received the ICI–TKI combination. At baseline, 52.5% of patients had an NLR ≥ 3.2, and 32% had bone metastases. After a median follow-up of 26.8 months, the median OS and median PFS were 34.7 and 11.3 months, respectively. Meet-URO demonstrated effective prognostic stratification, identifying patient groups with markedly different outcomes (median OS 11.5-51.4 months; 3-year OS 26-66%; RMST 20.0-42.8 months). Compared to IMDC, Meet-URO showed a significantly better OS (c-index 0.675 vs 0.643; Δc = 0.032, *P* < .001) and PFS (c-index 0.60 vs 0.58; *P* < .001) prediction performance.

**Conclusions:**

Meet-URO demonstrated robust prognostic accuracy. Its integration into routine clinical practice and use as a stratification factor in clinical trials may support more personalized treatment strategies and enhance clinical trial design.

Implications for Practice In patients with metastatic renal cell carcinoma receiving first-line immune-based combinations, the Meet-URO score provided more refined prognostic stratification than the IMDC model in a large international real-world cohort.

By integrating IMDC risk category with neutrophil-to-lymphocyte ratio and bone metastases, this simple clinical tool may improve outcome estimation, support patient counseling, and enhance risk stratification in clinical trials. Its use may be particularly relevant for patients classified as intermediate- or poor-risk by IMDC, in whom prognosis remains especially heterogeneous.

## Introduction

The advent of immune checkpoint inhibitors (ICIs) has transformed the therapeutic landscape of metastatic renal cell carcinoma (mRCC), offering significant survival and response benefits compared to tyrosine kinase inhibitors (TKIs) alone, particularly when used as first-line immune-based combinations.^1^

According to the updated 2024 European Society for Medical Oncology^2^ and 2025 European Association of Urology (EAU)^3^ clinical practice guidelines, combination treatment strategies for mRCC consist of dual ICIs, such as nivolumab plus ipilimumab, or ICI combined with TKIs, including nivolumab plus cabozantinib, pembrolizumab plus lenvatinib, pembrolizumab plus axitinib, toripalimab plus axitinib, or avelumab plus axitinib.

Despite these advances, treatment selection in mRCC remains complex due to the heterogeneity of clinical presentation, tumor biology, and therapeutic response. The International Metastatic RCC Database Consortium (IMDC) risk model has been the cornerstone prognostic tool in clinical trials and practice for nearly 2 decades, effectively predicting survival and guiding treatment decisions.^4^ However, some limitations have been recognized.

Primarily, the intermediate-risk group includes a large proportion of patients and represents a clinically heterogeneous population. Moreover, subsequent analyses have proposed a “very favorable” subgroup within the favorable-risk category incorporating metastatic site distribution as an additional prognostic factor^5^; this refinement is not part of the original IMDC model, which does not formally include metastatic site distribution among its variables.

In 2021, the Italian Meet-URO 15 study evaluated multiple prognostic factors, including patient characteristics, IMDC score, metastatic sites, and various immune-inflammatory indices, in 571 mRCC patients treated with second-line or later nivolumab, aiming to refine prognostic stratification in the immunotherapy era.^6^ Through rigorous statistical analyses, the study developed the Meet-URO score, integrating the IMDC score with neutrophil-to-lymphocyte ratio (NLR) and the presence of bone metastases, both established prognostic markers in mRCC.^6^

The Meet-URO score identifies 5 distinct prognostic groups and has demonstrated better accuracy in predicting overall survival (OS) and patient risk stratification compared to the IMDC score alone, for patients receiving second-line immunotherapy and TKIs.^6^^,^^7^ Its prognostic value was further validated in a prospective cohort of 306 IMDC intermediate- and poor-risk patients undergoing first-line nivolumab plus ipilimumab, confirming enhanced risk discrimination and clinical utility in the context of frontline immunotherapy.^8^^,^^9^ Additionally, a small retrospective study in pretreated patients receiving ICI–TKI combinations reported promising results supporting the Meet-URO score.^10^

Here, we present an external validation of the Meet-URO score’s prognostic performance in a large real-world multicentric cohort of mRCC patients treated with first-line immune-based combinations (ICI–ICI and ICI–TKI).

## Materials and methods

### Study design

The study was conducted as a multicentric, retrospective, observational real-world clinical audit at each participating center, following local regulations governing clinical audits. Data collection complied with anonymization standards and data transfer rules to protect patient confidentiality. All patients had previously provided informed consent consistent with institutional policies, thus negating the need for a formal ethical committee approval specifically for this external validation analysis of the Meet-URO score in mRCC patients receiving first-line immunotherapy combinations. The dataset was assembled by pooling anonymized clinical data originally collected for other research purposes, under protocols approved by local Institutional Review Boards or Ethics Committees, in full compliance with applicable data protection regulations.

### Study population

Eligible patients were adults (≥18 years) with a histologically confirmed diagnosis of mRCC, irrespective of histological subtype, who initiated first-line immune-based combination therapy between January 2019 and May 2024. Data collection and case identification were performed retrospectively across participating centers. Patients received first-line systemic therapy with either ICI–ICI or ICI–TKI combinations according to routine clinical practice. Treatment continued until radiological or clinical disease progression or unacceptable toxicity—based on the judgment of the treating medical oncologist—or until death or patient withdrawal.

Radiological assessments were performed based on RECIST 1.1 following local clinical practice, usually every 8-12 weeks. Baseline patient demographics, tumor characteristics, and treatment details were extracted from electronic patient records; prior nephrectomy was defined as surgical removal of the primary renal tumor before initiation of systemic therapy, including both radical nephrectomy for initially localized disease and cytoreductive nephrectomy in de novo metastatic patients. All data entry was conducted by trained investigators and monitored for completeness and accuracy by SER, VM, FC, and AS.

### Prognostic scores

The IMDC risk score is calculated using 6 variables (1 point per variable): time from diagnosis to systemic treatment, Karnofsky Performance Status, hemoglobin level, calcium corrected for serum albumin, neutrophil count, and platelet count, and stratifies patients into favorable- (0 points), intermediate- (1-2 points), and poor-risk (3-6 points) categories.

The Meet-URO score integrates the IMDC model by incorporating the NLR and the presence of bone metastases, categorizing patients into 5 prognostic groups ranging from group 1 (best prognosis) to group 5 (worst prognosis) (Tables S1 and S2). The Meet-URO score calculator is available online at https://proviso.shinyapps.io/Meet-URO15_score/.

### Study objectives and endpoints

The primary objective was to externally validate the Meet-URO score, while the secondary objective was to compare its prognostic performance against the IMDC score in predicting OS and progression-free survival (PFS).

The primary endpoint, OS, was defined as the time from initiation of first-line therapy to death from any cause; patients alive at the time of analysis were censored at their last follow-up date. The secondary endpoint, PFS, was assessed as an exploratory measure and defined as the time from treatment start to radiological or clinical disease progression or death, whichever occurred first. Disease response was evaluated referring to RECIST 1.1 as determined by local investigators.

In addition, the restricted mean survival time (RMST), representing the average time to event (death or progression) from baseline to the end of follow-up, was calculated to provide a complementary, model-free estimate of survival.

### Statistical analysis

Baseline characteristics were summarized using medians and interquartile ranges for continuous variables, and frequencies with percentages for categorical variables. OS and PFS for each Meet-URO and IMDC risk group were estimated using Kaplan–Meier curves, with group differences assessed by the log-rank test. Cox proportional hazards models were applied to estimate hazard ratios (HRs) and 95% CIs for survival outcomes. Logistic regression analyzed binary endpoints such as overall response rate and disease control rate. Median follow-up time was calculated using the reverse Kaplan–Meier method.

To evaluate and compare the prognostic performance of the Meet-URO and IMDC scores, Harrell’s concordance index (c-index) was estimated using a paired non-parametric bootstrap approach (1000 resamples), accounting for the correlation between predictions derived from the same patients. The 95% CI was calculated using the percentile method.

To provide a comprehensive assessment of model performance, we additionally evaluated model fit using Akaike’s Information Criterion (AIC) and log-likelihood values. Lower AIC and higher log-likelihood values were interpreted as indicating improved model fit.

To assess clinical reclassification performance, the net reclassification improvement (NRI) was calculated at 24 months for OS and 12 months for PFS using category-based reclassification with prespecified risk thresholds. Confidence intervals for NRI were obtained via bootstrap resampling (1000 iterations).

To address potential concerns regarding model granularity, analyses were performed using both the original 5-category Meet-URO score and a simplified 3-category version, and results were compared with the 3-category IMDC model.

Finally, an additional multivariable Cox regression model including IMDC category, neutrophil-to-lymphocyte ratio (NLR ≥3.2), and bone metastases was performed to evaluate the independent prognostic contribution of individual Meet-URO components and assess potential collinearity.

Calibration of both scores at 24 months was assessed using flexible parametric survival models (stpm2) based on linear predictors derived from Cox models incorporating categorical risk groups. Calibration plots, generated with pmcalplot, displayed observed vs predicted risks grouped by quantiles; the reference category was included in the models but did not appear as a separate point on the plots, as it defines baseline risk.

To assess clinical utility, decision curve analysis (DCA) was performed using predicted 24-month OS probabilities from Cox models. Net benefit was compared across a range of threshold probabilities between the Meet-URO and IMDC models.

All analyses were conducted by a professional biostatistician (A.S.) using Stata version 16.0 (StataCorp LLC).

## Results

### Patient characteristics and Meet-URO score distribution

A total of 1418 patients receiving first-line immune-based combinations between January 2019 and May 2024 were enrolled from 31 centers, including 27 in the UK, 2 in the Czech Republic, 1 in Turkey, and 1 in China. Patient characteristics for the overall cohort and stratified by the Meet-URO score are summarized in Table 1.

**Table 1.** Patients’ characteristics in the overall population and according to the Meet-URO score.

Characteristics	Overall population (*N* = 1418)	Meet-URO score prognostic group (*N*, %)					*P*-value
		1 (177, 12.4%)	2 (387, 27.3%)	3 (313, 22.1%)	4 (428, 30.2%)	5 (113, 8.0%)	
**Median age, years (IQR)**	62.1 (56.0-69.0)	58.7 (50.9-65.8)	62.2 (54.2-70.4)	64.2 (55.5-70.9)	62.3 (55.2-69.6)	61.0 (54.5-68.8)	<.001
**KPS**							<.001
** 80-100%**	1237 (87.2)	175 (98.9)	368 (95.1)	285 (91.1)	342 (79.9)	67 (59.3)	
** < 80%**	181 (12.8)	2 (1.1)	19 (4.9)	28 (8.9)	86 (20.1)	46 (40.7)	
**Gender**							.50
** Male**	1022 (72.1)	123 (69.5)	291 (75.2)	218 (69.7)	308 (72.0)	82 (72.6)	
** Female**	396 (27.9)	54 (30.5)	96 (24.8)	95 (30.3)	120 (28.0)	31 (27.4)	
**Metastatic at diagnosis**							<.001
** Yes**	906 (63.9)	56 (31.6)	205 (53.0)	195 (62.3)	350 (81.8)	100 (88.5)	
** No**	512 (36.1)	121 (68.4)	182 (47.0)	118 (37.7)	78 (18.2)	13 (11.5)	
**Nephrectomy**							<.001
** Yes**	739 (52.1)	151 (85.3)	264 (68.2)	170 (54.3)	136 (31.8)	18 (15.9)	
** No**	679 (47.9)	26 (14.7)	121 (31.8)	143 (45.7)	292 (68.2)	95 (84.1)	
**Histologic subtype**							.001
** Clear cell**	1213 (85.5)	163 (92.1)	340 (87.9)	271 (86.6)	352 (82.2)	87 (77.0)	
** Non-clear cell**	205 (14.5)	14 (7.9)	47 (12.1)	42 (13.4)	76 (17.8)	26 (23.0)	
**IMDC score**							<.001
** Favorable**	273 (19.3)	177 (100.0)	96 (24.8)	0	0	0	
** Intermediate**	753 (53.1)	0	291 (75.2)	313 (100.0)	149 (34.8)	0	
** Poor**	392 (27.6)	0	0	0 (0)	279 (65.2)	113 (100)	
**NLR**							<.001
** < 3.2**	673 (47.5)	177 (100.0)	291 (75.2)	104 (33.2)	101 (23.6)	0	
** ≥ 3.2**	745 (52.5)	0	96 (24.8)	209 (66.8)	327 (76.4)	113 (100)	
**Bone metastases**							<.001
** No**	963 (67.9)	146 (82.5)	369 (95.4)	209 (66.8)	239 (55.8)	0	
** Yes**	455 (32.1)	31 (17.5)	18 (4.6)	104 (33.2)	189 (44.2)	113 (100)	
**First-line therapy**							<.001
** ICI**–**ICI**	770 (54.3)	51 (28.8)	215 (55.6)	188 (60.1)	252 (58.9)	64 (56.6)	
** ICI**–**TKI**	648 (45.7)	126 (71.1)	172 (44.4)	125 (39.9)	176 (41.1)	49 (43.4)	
**Immune-combinations**							
** Nivo + Ipi**	770 (54.3)	51 (28.8)	215 (55.6)	188 (60.1)	252 (58.9)	64 (56.6)	<.001
** Nivo + Cabo**	13 (0.9)	1 (0.6)	5 (1.3)	3 (1.0)	3 (0.7)	1 (0.9)	
** Pembro + Axi**	111 (7.8)	20 (11.3)	32 (8.2)	16 (5.1)	36 (8.4)	7 (6.2)	
** Pembro + Lenva**	185 (13.0)	12 (6.8)	41 (10.6)	47 (15.0)	63 (14.7)	22 (19.5)	
** Ave + Axi**	338 (23.9)	93 (52.5)	94 (24.3)	58 (18.5)	74 (17.3)	19 (16.8)	
** Nivo + Axi**	1 (0.1)	0	0	1 (0.3)	0	0	

Abbreviations: Ave, avelumab; Axi, axitinib; Cabo, cabozantinib; ICI, immune checkpoint inhibitor; IMDC, International Metastatic RCC Database Consortium; Ipi, ipilimumab; IQR, interquartile range; Lenva, lenvatinib; *N*, number of patients; Nivo, nivolumab; NLR, neutrophil-to-lymphocyte ratio; Pembro, pembrolizumab; TKI, tyrosine kinase inhibitor.

The median age was 62 years, with males comprising 72% of the population. Clear-cell RCC accounted for 86% of cases, 52% of patients had undergone prior nephrectomy, and 64% were metastatic at diagnosis. As expected, 95.9% of patients initially diagnosed with localized disease had undergone prior radical nephrectomy, whereas 27.4% of de novo metastatic patients underwent nephrectomy.

Regarding first-line systemic treatments, 54% received dual ICI therapy (nivolumab plus ipilimumab), while 46% were treated with ICI–TKI combinations, most commonly avelumab plus axitinib (24%).

According to the IMDC risk classification, 19% of patients were favorable-risk, 53% intermediate-risk, and 28% poor-risk. At baseline, 53% had an elevated neutrophil-to-lymphocyte ratio (NLR ≥ 3.2), and 32% presented with bone metastases.

The Meet-URO score stratified patients into 5 prognostic groups distributed as follows: group 1 (13%), group 2 (27%), group 3 (22%), group 4 (30%), and group 5 (8%). Compared to group 1, patients in groups 4 and 5 showed a marked increase in poor performance status (41% vs 1%), higher rates of metastatic disease at diagnosis (89% vs 32%), and lower rates of prior radical surgery (16% vs 85%) and clear-cell histology (77% vs 92%) (Table 1).

### Concordance and stratification between Meet-URO and IMDC scores

According to the IMDC classification, the favorable-risk group corresponded to Meet-URO groups 1 and 2, representing 65% and 35% of that category, respectively. The intermediate-risk IMDC group was further subdivided into Meet-URO groups 2, 3, and 4, accounting for 39%, 41%, and 20%, respectively. The poor-risk IMDC group comprised Meet-URO groups 4 and 5, representing 71% and 29%, respectively (Figure S1).

Conversely, within the Meet-URO score categories, all patients in group 1 were classified as IMDC favorable-risk. Group 2 patients were split between IMDC favorable-risk (25%) and intermediate-risk (75%). Group 3 exclusively included IMDC intermediate-risk patients. Group 4 comprised both IMDC intermediate-risk (35%) and poor-risk (65%) patients, while all patients in group 5 fell within the IMDC poor-risk category (Table 1).

### Prognostic discrimination of the Meet-URO score for overall survival

With a median follow-up of 26.8 months (range: 25.7-28.6), the median OS and PFS were 34.7 and 11.3 months, respectively.

The Meet-URO score demonstrated good discriminatory power for predicting OS, with a c-index of 0.675. It effectively stratified patients into prognostic groups exhibiting distinct median OS, 3-year OS rates (3 y-OS), and RMST.

**Figure 1.**
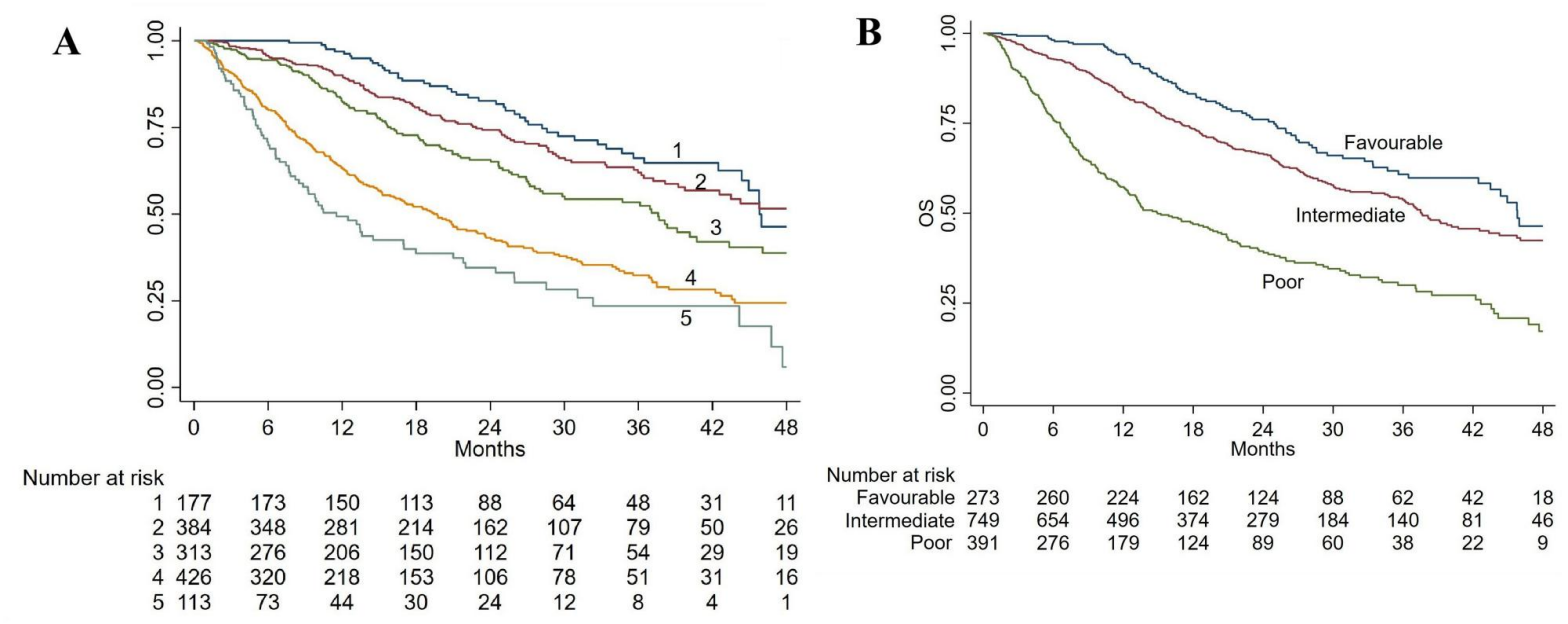
Overall survival (OS) according to the 5-category Meet-URO score (A) and the 3-category IMDC risk group (B).

Median OS values for the Meet-URO groups showed a clear risk gradient: 45.8 and 51.4 months for the most favorable groups (1 and 2), 37.6 months for group 3, 19.7 months for group 4, and a markedly lower 11.5 months for group 5 (Table 2 and Figure 1A). Similarly, 3 y-OS declined significantly across groups: 66% (group 1), 62% (group 2), 53% (group 3), 32% (group 4), and 26% (group 5) (Table 2 and Figure 1A).

**Table 2.** Univariable analysis of Meet-URO and IMDC scores on OS.

Score	*N* (%)	HR (95% CI)	*P*-value	mOS (mo)	RMST (mo)	3y-OS (%)	c-index (95% CI)
**Meet-URO**							
** 1**	177 (12.4)	1.00 (ref)		45.8	42.8	66	
** 2**	387 (27.3)	1.23 (0.88-1.72)	.23	51.4	40.8	62	0.675 (0.654-0.696)
** 3**	313 (22.1)	1.74 (1.24-2.44)	.001	37.6	35.9	53	
** 4**	428 (30.2)	3.31 (2.42-4.51)	<.001	19.7	26.3	32	
** 5**	113 (8.0)	4.82 (3.35-6.94)	<.001	11.5	20.0	26	
**Meet-URO (3 categories)**							
** 1-2**	564 (39.7)	1.00 (ref)		48.6	41.4	63	
** 3-4**	741 (52.3)	2.22 (1.84–2.68)	<.001	25.4	30.2	41	0.641 (0.621-0.661)
** 5**	113 (8.0)	4.18 (3.17–5.52)	<.001	11.5	20.0	26	
**IMDC**							
** Favorable**	273 (19.3)	1.00 (ref)		45.8	40.9	61	
** Intermediate**	753 (53.1)	1.40 (1.09-1.79)	.007	37.6	36.7	54	0.643 (0.621-0.664)
** Poor**	392 (27.6)	3.23 (2.51-4.16)	<.001	14.9	23.8	30	

Abbreviations: HR, hazard ratio; IMDC, international metastatic renal cell carcinoma database consortium; mOS, median overall survival; *N*, number of patients; *NR*, not reached; 3 y-OS, overall survival rate at 3 years; RMST, restricted mean survival time calculated at the maximum follow-up for all groups of 58 months. Model performances: Meet-URO (5 categories): c-index 0.675; AIC 7556.3; NRI 0.063 (95% CI 0.024-0.103). Meet-URO (3 categories): c-index 0.641; AIC 7587.5; NRI −0.163 (95% CI −0.217 to −0.113). IMDC: c-index 0.643; AIC 7592.4.

These differences were statistically significant (*P *< .001), with HRs for death rising progressively from 1.23 in group 2 to 4.82 in group 5, using group 1 as the reference (Table 2).

RMST values further underscored this distinction, measuring 42.8, 40.8, 35.9, 26.3, and 20 months from groups 1 through 5, respectively (Table 2).

Median survival estimates in lower-risk groups should be interpreted cautiously given the relatively short follow-up; therefore, RMST and fixed-time survival probabilities are provided as complementary and more robust measures.

In a multivariable Cox regression including IMDC category, NLR (≥3.2), and bone metastases, all components of the Meet-URO score retained independent prognostic value (Table S3).

### Prognostic discrimination of the Meet-URO score for progression-free survival

The Meet-URO score demonstrated also a sufficient discriminatory power for predicting PFS, with a c-index of 0.602.

**Figure 2.**
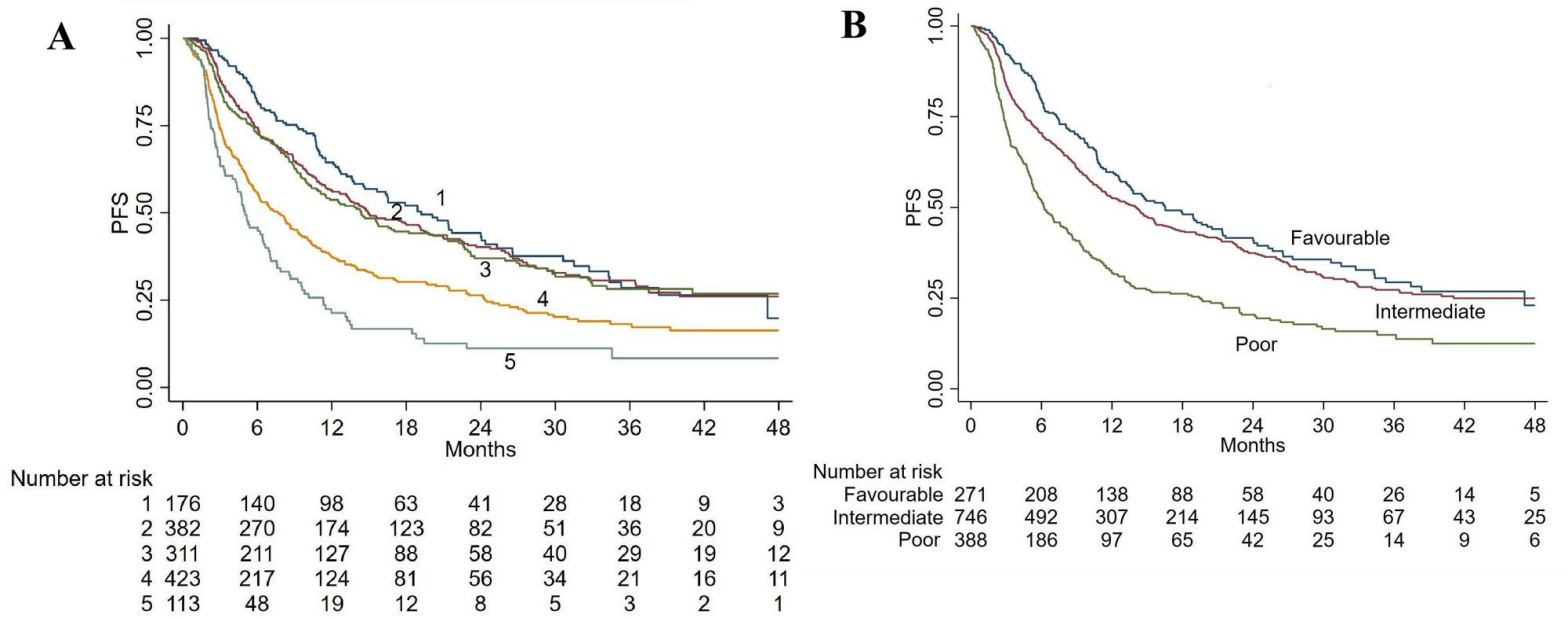
Progression-free survival (PFS) according to the 5-category Meet-URO score (A) and the 3-category IMDC risk group (B).

PFS results for the Meet-URO groups showed a good risk gradient in terms of mPFS (range: 19.2-5.0 months), 1-year PFS rates (1 y-PFS) (range: 64.4-21.3%) and RMST (range: 24.9-10.7 months) from groups 1 through 5, respectively (Table 3 and Figure 2A). These differences were statistically significant (*P *< .001) only for the worst groups, with HRs for death rising progressively from 1.16 in group 2 to 2.87 in group 5, using group 1 as the reference (Table 3 and  Figure 2A).

**Table 3.** Univariable analysis of Meet-URO and IMDC scores on PFS.

Score	*N* (%)	HR (95% CI)	*P*-value	mPFS (mo)	RMST (mo)	1y-PFS (%)	c-index (range)
**Meet-URO**							
** 1**	177 (12.4)	1.00 (ref)		19.2	24.9	64.4	
** 2**	387 (27.3)	1.16 (0.91-1.46)	.23	14.9	23.4	56.4	0.602 (0.583-0.620)
** 3**	313 (22.1)	1.20 (0.94-1.54)	.14	14.4	23.1	53.7	
** 4**	428 (30.2)	1.89 (1.50-2.37)	<.001	7.7	16.4	37.4	
** 5**	113 (8.0)	2.87 (2.16-3.81)	<.001	5.0	10.7	21.3	
**Meet-URO (3 categories)**							
** 1-2**	564 (39.7)	1.00 (ref)		16.5	23.9	58.8	
** 3-4**	741 (52.3)	1.41 (1.22-1.62)	<.001	9.8	19.6	44.2	0.580 (0.563-0.597)
** 5**	113 (8.0)	2.58 (2.04-3.25)	<.001	5.0	10.7	21.3	
**IMDC**							
** Favorable**	273 (19.3)	1.00 (ref)		16.5	24.2	59.7	
** Intermediate**	753 (53.1)	1.18 (0.98-1.41)	.082	14.1	22.2	52.7	0.582 (0.564-0.600)
** Poor**	392 (27.6)	2.01 (1.65-2.44)	<.001	6.2	14.3	32.0	

Abbreviations: HR, hazard ratio; IMDC, international metastatic renal cell carcinoma database consortium; RMST, restricted mean survival time; mOS, median overall survival; *N,* number of patients; NR, not reached;1 y-PFS, progression-free survival at 1 year.

### Comparative prognostic performance and clinical utility of the Meet-URO and IMDC scores

When comparing prognostic performance, the Meet-URO score demonstrated superior discrimination for OS compared with the IMDC model (c-index: 0.675 vs 0.643). The absolute difference in c-index was Δc = 0.032 (95% CI 0.019-0.046; *P* < .001), indicating a modest but statistically significant improvement in prognostic accuracy (Table S4). To provide a comprehensive comparison of prognostic performance, we complemented the c-index analysis with model fit indices and net reclassification metrics. Also model fit indices strongly favored Meet-URO on AIC and log-likelihood (Table S4), and net reclassification analysis at 24 months demonstrated a significant improvement of Meet-URO score in patient risk classification (NRI 0.063, 95% CI 0.024-0.103), corresponding to a 6.3% net improvement in correct risk classification at 24 months.

Differences in median OS, 3 y-OS, and RMST were more pronounced across Meet-URO risk groups than those defined by the IMDC score (Table 2 and  Figure 1).

Calibration plots at 24 months showed good agreement between predicted and observed event probabilities for both models (Figure S2). The Meet-URO model, based on its 5-category stratification, displayed strong calibration across all prognostic groups, with minimal deviation from observed outcomes. Similarly, the IMDC model exhibited adequate calibration, with predicted probabilities closely aligned with observed event rates in both risk categories. For both models, the reference group contributed to baseline risk estimation but is not shown as a separate point in the plots.

In the DCA for 24-month OS, the Meet-URO score provided a higher net clinical benefit than the IMDC model at threshold probabilities above 20%, indicating improved clinical utility, particularly in identifying patients with at least intermediate-risk disease who are more likely to benefit from treatment (Figure S3).

When restricting the comparison to 3-category models (Table S4), the discriminative performance of Meet-URO and IMDC was comparable (c-index 0.641 vs 0.643, respectively). Net reclassification did not favor the simplified Meet-URO version (NRI −0.163, 95% CI −0.217 to −0.113).

For PFS, the 5-category Meet-URO score (Table S5) showed higher discrimination than IMDC (c-index 0.602 vs 0.582; Δc = 0.020, 95% CI 0.009-0.032; *P* < .001). The 3-category comparison showed no meaningful advantage, whereas the original 5-category structure demonstrated a modest but significant improvement also in reclassification (NRI 0.038, 95% CI 0.008-0.069).

### Consistent prognostic performance of Meet-URO score across immunotherapy regimens

The Meet-URO score maintained strong prognostic discrimination across both immunotherapy combination types (ICI–ICI and ICI–TKI), consistently outperforming the IMDC score regardless of treatment regimen. Notably, both models demonstrated better prognostic performance in patients treated with ICI–TKI combinations compared to those receiving ICI–ICI therapy (Table S6).

## Discussion

Accurate prognostic classification has become increasingly important in the evolving treatment landscape of mRCC, supporting clinical trial design, patient counseling, and treatment decision-making. While the IMDC score remains the most widely used prognostic model in both clinical practice and trials, its limitations have become more apparent, particularly in the era of immunotherapy-based combinations.

A major drawback of the IMDC score is its intermediate-risk group, which includes more than half of all patients and represents a clinically and biologically heterogeneous population. Post hoc analyses from pivotal trials such as KEYNOTE-426 and CheckMate 214 have shown significant variability in survival outcomes among intermediate-risk patients, reflecting underlying differences in tumor biology and patient characteristics.^11^^,^^12^

Emerging evidence suggests that inflammation-based markers, such as the NLR, offer improved prognostic insight over absolute immune cell counts by capturing the balance between pro- and anti-tumor immune responses.^6^ Likewise, the presence and pattern of metastatic sites, particularly bone metastases, have been associated with distinct tumor microenvironments and differential immunotherapy outcomes.^13^

The Meet-URO score builds on these principles by integrating the IMDC risk group, NLR, and the presence of bone metastases, with each component weighted according to its independent prognostic impact.^6^ Unlike the equal weighting used in the IMDC model, the Meet-URO score applies differential weights to define 5 prognostic categories, enabling a more nuanced risk stratification. Our current analysis, one of the largest real-world validations of a refined prognostic model in mRCC, confirmed the good prognostic discrimination of the Meet-URO score in first-line immunotherapy-treated patients—also in comparison with the IMDC model. While the absolute difference in c-index was modest, this finding aligns with prior studies and highlights the consistency and generalizability of the Meet-URO score across different settings (Table S7).^6–10^ Importantly, the Meet-URO score was also validated in a large-scale ambispective analysis of 1255 Italian patients treated in the first-line setting, further confirming its reliability in routine clinical use.^14^

In our analysis, the Meet-URO score demonstrated a clear gradient of outcomes across its 5 risk groups, with median OS ranging from 11.5 months (group 5) to nearly 48 months (groups 1–2), and corresponding 3 y-OS from 26% to 66%, a meaningful differentiation for clinical practice.

In addition to its overall stratification power, the score showed value in refining prognosis within each IMDC category. For example, the IMDC favorable group was split into 2 subgroups, “very-favorable” (group 1) and “favorable” (group 2), with slightly different long-term survival outcomes (3 y-OS: 66% vs 62%). While the difference may appear modest, longer follow-up could accentuate these trends.

In the IMDC poor-risk group, the Meet-URO score identified a lower-risk subgroup (group 4, “intermediate-poor”) with a median OS of 19.7 months, significantly higher than the 11.5 months observed in group 5 (“very-poor”). Notably, group 4 outcomes more closely resembled those of the IMDC intermediate-risk category.

Specifically, the Meet-URO score subdivided the heterogeneous IMDC intermediate-risk group into 3 distinct categories, “favorable-intermediate,” “real-intermediate,” and “poor-intermediate,” with median OS ranging from 19.7 to 51.4 months and 3 y-OS from 32% to 62%.

While other inflammation-based prognostic models, such as the Lung Immune Prognostic Index and the modified Glasgow Prognostic Score, have shown promising results in mRCC,^15^^,^^16^ they rely on biomarkers like lactate dehydrogenase, C-reactive protein, and albumin. These markers, while informative, are often affected by non-cancer-related conditions and may not be routinely assessed in oncology clinics, limiting their practical applicability.

By contrast, the Meet-URO score incorporates readily available clinical parameters with established oncologic relevance and is accessible via a user-friendly web calculator. These features make it an attractive tool for daily practice, where the score may prove particularly useful in clinical situations where the IMDC model provides limited prognostic resolution. For example, in patients classified as IMDC intermediate-risk—a category encompassing more than half of patients with metastatic RCC—the Meet-URO score may help refine prognostic stratification and better contextualize expected outcomes when discussing first-line treatment options. Likewise, among patients categorized as IMDC poor-risk, the Meet-URO score may help distinguish those with extremely unfavorable prognosis from those with relatively preserved outcomes and, at the same time, it may identify the “very favorable” subgroup within IMDC good-risk patients, supporting more nuanced discussions on treatment intensity and the supposed benefit of combination therapy.^17^ In addition, the score may prove useful in routine clinical practice by offering more individualized prognostic estimates based on systemic inflammatory markers and metastatic site distribution, 2 features not captured by the IMDC model alone.

From a research perspective, it would also hold promise as a stratification factor in clinical trials. The limitations of the IMDC model in adequately separating risk groups can dilute observed treatment effects or introduce imbalances in randomized trials; the use of the Meet-URO score could therefore improve the interpretability of trial outcomes, especially in studies testing biomarker-driven or risk-adapted approaches. However, the use of a 5-category stratification model in randomized trials may increase design complexity and potentially impact required sample size, particularly if event numbers within individual strata become limited. Therefore, while the enhanced prognostic granularity of the Meet-URO score may reduce within-group heterogeneity, its incorporation into trial stratification schemes should be carefully balanced against statistical efficiency and feasibility considerations.

That said, the present study has several limitations. Its retrospective and observational nature introduces the potential for bias, including selection bias, confounding factors, and non-standardized disease assessments. Another limitation is the lack of detailed information regarding the indication and timing of nephrectomy, as the database did not allow discrimination between cytoreductive nephrectomy, nephrectomy performed for initially localized disease prior to metastatic relapse, or nephrectomy performed in combination with metastasectomy; this information would have been of interest, given the important role nephrectomy still plays in the management of selected patients in contemporary clinical practice.^18^ In addition, follow-up was relatively short for the most favorable Meet-URO groups, potentially underestimating long-term differences.

Future directions include prospective analyses assessing whether the Meet-URO score can guide treatment selection within each risk group. Additionally, dedicated randomized studies could help test the score’s predictive value across therapeutic strategies (ICI–ICI vs ICI–TKI vs TKI monotherapy) and settings; as adjuvant immunotherapy becomes more widely adopted, future studies should prospectively capture prior treatment exposure and evaluate its potential interaction with clinical prognostic models. Also, although this cohort included both clear-cell (85.5%) and non–clear-cell RCC (14.5%), histology-specific evaluation of survival outcomes and comparative prognostic performance of Meet-URO vs IMDC was beyond the scope of the present external validation and is being pursued in a dedicated analysis.

While the Meet-URO score improves prognostic stratification compared with the IMDC model using readily available clinical variables, it likely reflects the upper limit of prognostic refinement achievable with clinical factors alone. Future progress in risk stratification and development of available scores will depend on integrating clinical models with tissue-based biomarkers and genomic classifications.^12^

## Conclusions

The Meet-URO score is a reproducible, practical, and clinically meaningful tool that refines prognostic classification in patients with mRCC, offering enhanced risk stratification beyond the established IMDC model, and is well-suited to the current disease setting with today’s available treatments.

In this large-scale, real-world external validation, the Meet-URO score demonstrated consistent prognostic accuracy across a contemporary first-line treatment landscape, including both ICI–ICI and ICI–TKI combinations. By incorporating inflammation-based markers and metastatic site distribution, the score provides a more nuanced, biologically informed stratification of patient outcomes.

Given its strong discriminatory performance, ease of use, and applicability across different systemic therapies, the Meet-URO score holds promise for routine clinical use and may serve as a valuable tool for stratification and design in future clinical trials.

## Supplementary Material

oyag203_Supplementary_Data

## Data Availability

The data underlying this article are not publicly available because of institutional, ethical, and privacy restrictions related to the use of multicenter retrospective clinical data. De-identified data may be made available from the corresponding author upon reasonable request, subject to approval by the participating institutions and in accordance with applicable data protection regulations.
