# Supplementary material for: Refining prognosis in advanced renal cell carcinoma: international real-world validation of the Meet-URO score in first-line immunotherapy combinations
Source: Oncologist. 2026 May 20;31(7):oyag203. doi: 10.1093/oncolo/oyag203 (PMC13296786; doi:10.1093/oncolo/oyag203)
Supplement: oyag203_Supplementary_Data [file oyag203_supplementary_data.zip › Supplementary Tables.docx]

**Supplementary Table 1.** Meet-URO score calculation

| **Component** | **Category / cut-off** | **Points** |
| --- | --- | --- |
| IMDC risk group | Favorable | 0 |
|  | Intermediate | 3 |
|  | Poor | 6 |
| Neutrophil-to-lymphocyte ratio (NLR) | < 3.2 | 0 |
|  | ≥ 3.2 | 2 |
| Bone metastases | Absent | 0 |
|  | Present | 1 |

**Supplementary Table 2.** Meet-URO score risk groups

| **Meet-URO risk group** | **Total score** | **Definition (as in the paper)** |
| --- | --- | --- |
| Group 1 | 0–1 | No prognostic factors, or bone metastases only |
| Group 2 | 2–3 | NLR ≥ 3.2 or IMDC intermediate |
| Group 3 | 4–5 | IMDC intermediate and (bone metastases or NLR ≥ 3.2) |
| Group 4 | 6–8 | IMDC intermediate + bone metastases + NLR ≥ 3.2 or IMDC poor alone or IMDC poor + (bone metastases or NLR ≥ 3.2) |
| Group 5 | 9 | NLR ≥ 3.2 + IMDC poor + bone metastases |

*NLR* neutrophil-to-lymphocyte ratio, *IMDC* International Metastatic RCC Database Consortium, *N* number of patients, *mOS* median overall survival, *HR* hazard ratio, *CI* confidence interval, *Ref* reference group, *NR* not reached.

**Supplementary Table 3**. Multivariable Cox regression assessing the independent prognostic impact of the Meet-URO score components on overall survival.

| **Variabile** | **HR** | **95% CI** | **p-value** |
| --- | --- | --- | --- |
| IMDC intermediate vs favorable | 1.26 | 0.98–1.61 | 0.07 |
| IMDC poor vs favorable | 2.48 | 1.91–3.22 | <0.001 |
| NLR ≥ 3.2 | 1.8 | 1.51–2.15 | <0.001 |
| Bone metastases | 1.26 | 1.06–1.49 | 0.008 |

**Supplementary Table 4.** Comparative Model Performance Metrics (OS)

| **Model** | **Categories** | **C-index (95% CI)** | **AIC** | **Log-likelihood** | **NRI (95% CI)** |
| --- | --- | --- | --- | --- | --- |
| IMDC | 3 | 0.643 (0.621–0.664) | 7592.4 | -3794.2 | Reference |
| Meet-URO | 3 | 0.641 (0.621–0.661) | 7587.5 | -3791.8 | -0.163 (-0.217 to -0.113) |
| Meet-URO | 5 | 0.675 (0.654–0.696) | 7556.3 | -3774.1 | 0.063 (0.024–0.103) |

AIC: Akaike Information Criterion; NRI: net reclassification index

**Supplementary Table 5.** Comparative Model Performance Metrics (PFS)

| **Model** | **Categories** | **C-index (95% CI)** | **AIC** | **Log-likelihood** | **NRI (95% CI)** |
| --- | --- | --- | --- | --- | --- |
| IMDC | 3 | 0.582 (0.564–0.600) | 11740.6 | -5868.3 | Reference |
| Meet-URO | 3 | 0.580 (0.563–0.597) | 11742.9 | -5869.4 | −0.010 (−0.062–0.043) |
| Meet-URO | 5 | 0.602 (0.583–0.620) | 11721.6 | -5856.8 | 0.038 (0.008–0.069) |

AIC: Akaike Information Criterion; NRI: net reclassification index

**Supplementary Table 6.** Univariable analysis of Meet-URO and IMDC scores on OS according to immune-based combination.

| **Score** | **N (%)** | **HR (95% CI)** | **p value** | **mOS (mo)** | **c-index (range)** |
| --- | --- | --- | --- | --- | --- |
| **ICI-TKI** |  |  |  |  | 0.72  (0.65-0.79) |
| **Meet-URO** |  |  |  |  |  |
| 1 | 51 (6.7) | Ref. |  | 48.6 |  |
| 2 | 215 (27.9) | 1.53 (0.95-2.46) | 0.078 | 42.6 |  |
| 3 | 188 (24.4) | 1.85 (1.12-3.06) | 0.016 | 37.7 |  |
| 4 | 252 (32.7) | 4.99 (3.24-7.69) | <0.001 | 17.1 |  |
| 5 | 64 (8.3) | 7.71 (4.58-12.98) | <0.001 | 9.3 |  |
| **IMDC** |  |  |  |  | 0.68  (0.59-0.77) |
| Favorable | 86 (11.2) | Ref. |  | 45.8 |  |
| Intermediate | 454 (59.0) | 1.61 (1.14-2.29) | 0.007 | 37.2 |  |
| Poor | 230 (29.8) | 4.25 (2.97-6.08) | <0.001 | 13.5 |  |
| **ICI-ICI** |  |  |  |  | 0.63  (0.56-0.70) |
| **Meet-URO** |  |  |  |  |  |
| 1 | 126 (19.4) | Ref. |  | 44.4 |  |
| 2 | 172 (26.5) | 0.86 (0.52-1.40) | 0.54 | 55.0 |  |
| 3 | 125 (19.3) | 1.31 (0.81-2.11) | 0.23 | 34.7 |  |
| 4 | 176 (27.2) | 2.08 (1.31-3.28) | 0.002 | 20.9 |  |
| 5 | 49 (7.6) | 2.88 (1.70-4.85) | <0.001 | 13.2 |  |
| **IMDC** |  |  |  |  | 0.61  (0.52-0.70) |
| Favorable | 187 (28.9) | Ref. |  | 44.4 |  |
| Intermediate | 299 (46.1) | 1.10 (0.77-1.57) | 0.61 | 37.6 |  |
| Poor | 162 (25.0) | 2.35 (1.63-3.39) | <0.001 | 16.7 |  |

*N* number of patients, *HR* hazard ratio, *mOS* median overall survival, *IMDC* international metastatic renal cell carcinoma database consortium, *NR* not reached.

**Supplementary Table 7.** Prognostic performance of the Meet-URO score compared with the IMDC score in mRCC patients in different therapeutic settings.

| **First name, year [Reference]** | **N** | **Therapy line** | **Study nature** | **Treatment type** | **IMDC score**  **c index** | **Meet-URO score**  **c index** |
| --- | --- | --- | --- | --- | --- | --- |
| Rebuzzi, 2021 [5] | 571 | 2^nd^ | Retrospective | Nivolumab | 0.64 | 0.69 |
| Rebuzzi, 2022 [6] | 174 | 2^nd^ - 3^rd^ | Retrospective | Cabozantinib | 0.57 | 0.64 |
| Rebuzzi, 2022 [7] | 306 | 1^st^ | Prospective | Nivolumab + Ipilimumab | 0.65 | 0.73 |
| He, 2024 [9] | 72 | 2^nd^ - 3^rd^ | Retrospective | ICI-TKI | 0.56 | 0.71 |
| Meet-URO 33 [13] | 1255 | 1^st^ | Prospective / Retrospective | ICI-ICI, ICI-TKI, TKI | 0.69 | 0.71 |
| International collaboration  (This study) | 1418 | 1^st^ | Retrospective | ICI-ICI, ICI-TKI | 0.64 | 0.68 |

*N* number of patients, *ICI* immune-checkpoint inhibitor, *TKI* tyrosine kinase inhibitor, *IMDC* international metastatic renal cell carcinoma database consortium.
