# Supplementary figures and images for: Refining prognosis in advanced renal cell carcinoma: international real-world validation of the Meet-URO score in first-line immunotherapy combinations
Source: Oncologist. 2026 May 20;31(7):oyag203. doi: 10.1093/oncolo/oyag203 (PMC13296786; doi:10.1093/oncolo/oyag203)

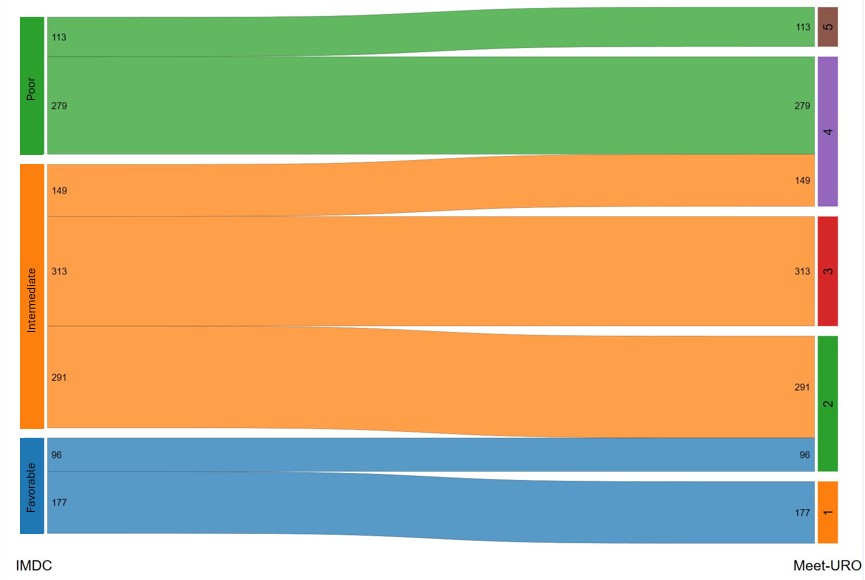

Supplement: oyag203_Supplementary_Data [file oyag203_supplementary_data.zip › Supplementary Figure 1.jpg]

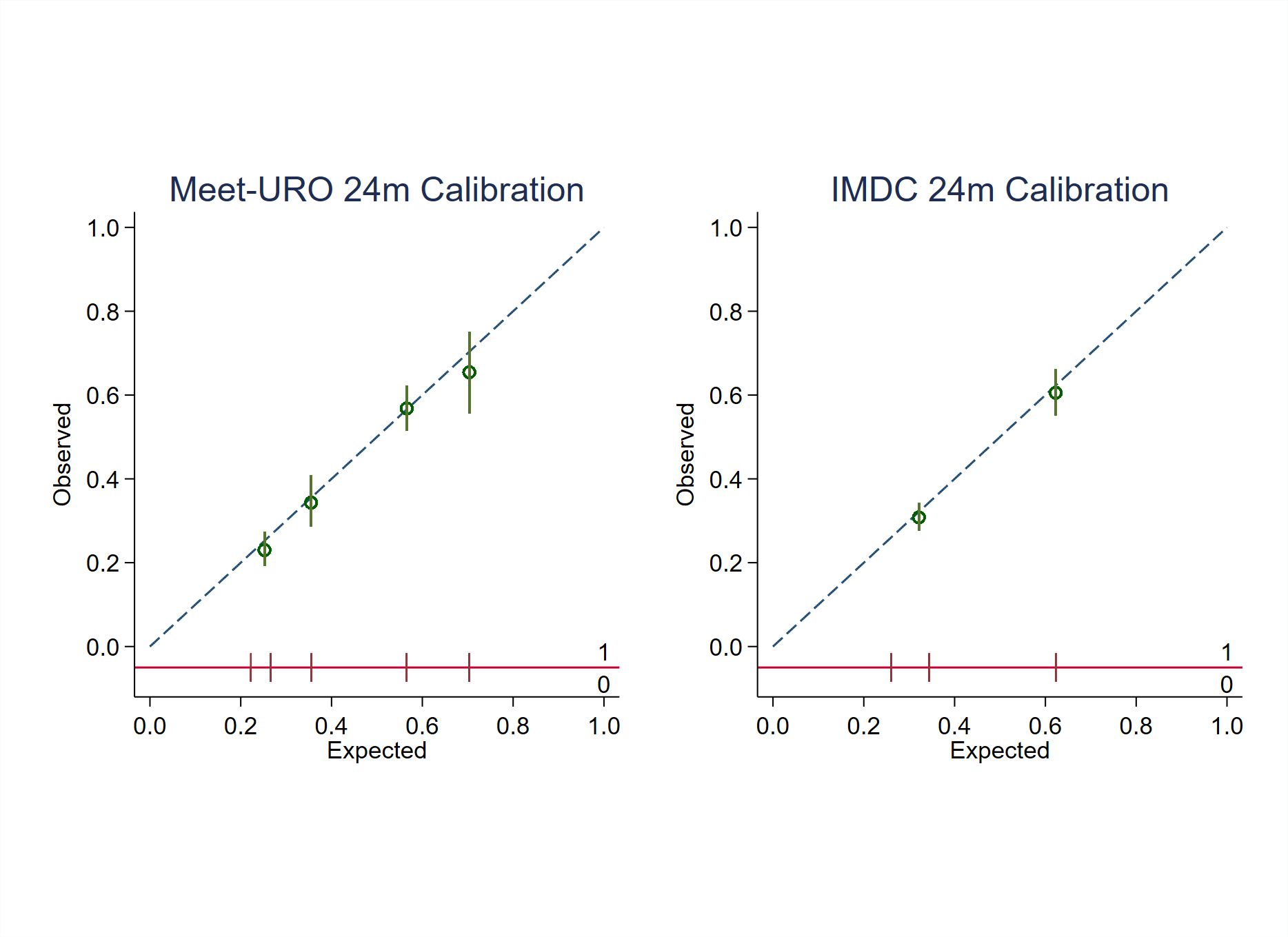

Supplement: oyag203_Supplementary_Data [file oyag203_supplementary_data.zip › Supplementary Figure 2.jpg]

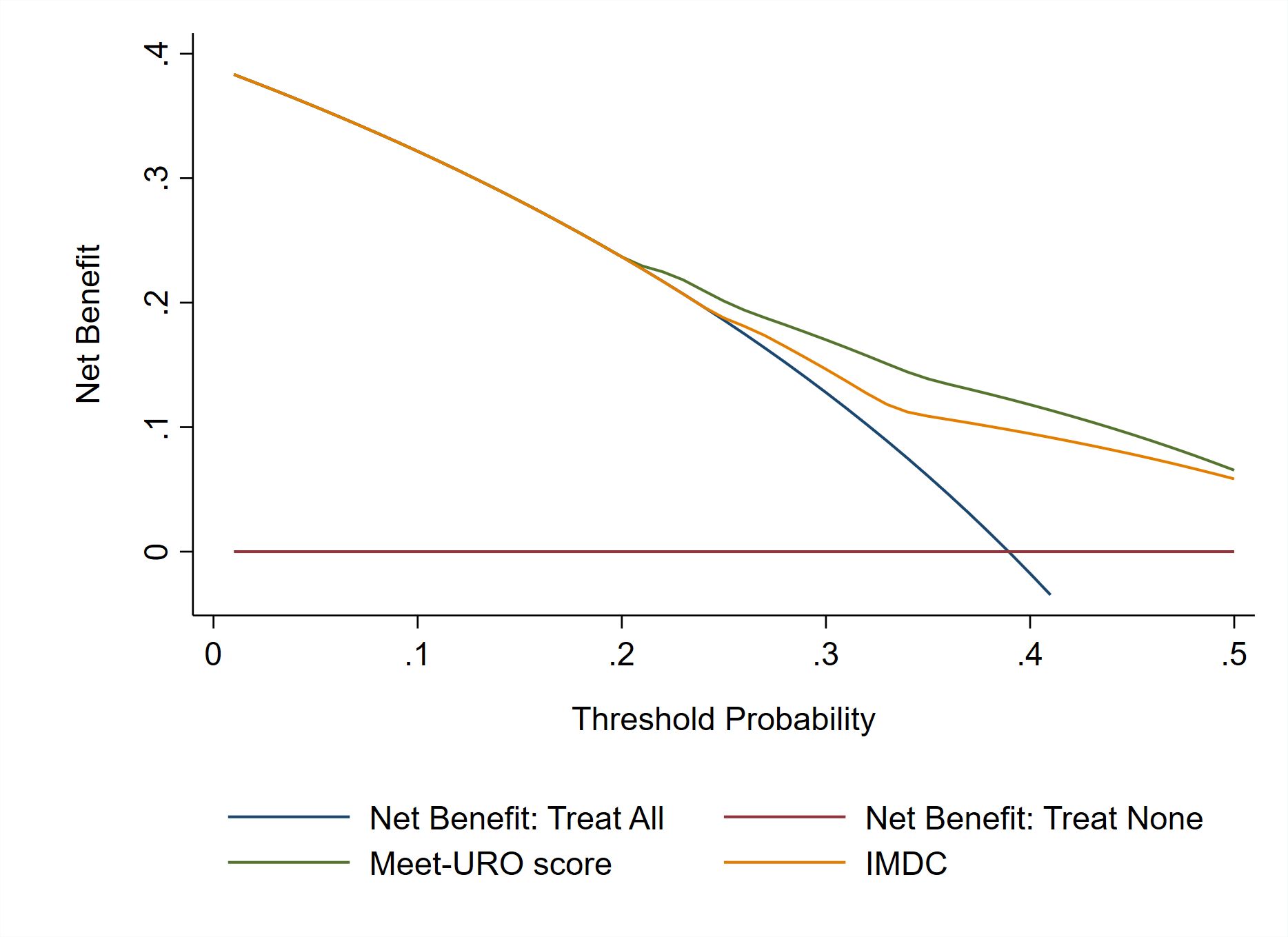

Supplement: oyag203_Supplementary_Data [file oyag203_supplementary_data.zip › Supplementary Figure 3.jpg]
